# Risk factors of NSAID‐exacerbated respiratory disease: A population‐based study

**DOI:** 10.1002/clt2.12296

**Published:** 2023-08-22

**Authors:** Alma Helevä, Aada Murtomäki, Heini Huhtala, Jean Bousquet, Annika Luukkainen, Jussi Karjalainen, Riikka Lemmetyinen, Jari Haukka, Paulus Torkki, Mikko Nuutinen, Sanna Toppila‐Salmi

**Affiliations:** ^1^ Skin and Allergy Hospital Helsinki University Hospital and University of Helsinki Hospital District of Helsinki and Uusimaa Helsinki Finland; ^2^ MD PhD Programme of the Faculty of Medicine University of Helsinki Helsinki Finland; ^3^ Faculty of Social Sciences Tampere University Tampere Finland; ^4^ Institute for Allergology Charité – Universitätsmedizin Berlin Corporate Member of Freie Universität Berlin and Humboldt‐Universität zu Berlin Berlin Germany; ^5^ Fraunhofer Institute for Translational Medicine and Pharmacology ITMP Allergology and Immunology Berlin Germany; ^6^ University Hospital of Montpellier Montpellier France; ^7^ Infectious Disease Unit South Karelia Central Hospital Lappeenranta Finland; ^8^ Allergy Centre Tampere University Hospital Tampere Finland; ^9^ Faculty of Medicine and Health Technology Tampere University Tampere Finland; ^10^ Department of Public Health University of Helsinki Helsinki Finland; ^11^ Haartman Institute Medicum University of Helsinki Helsinki Finland; ^12^ Department of Pulmonary Medicine Heart and Lung Center Helsinki University Hospital and University of Helsinki Helsinki Finland

**Keywords:** AERD, allergy, ASA‐intolerance, asthma, NERD

## Abstract

**Background:**

Asthma with NSAID‐exacerbated respiratory disease (NERD) is associated with uncontrolled or severe asthma. NERD patients are more prone to severe allergic reactions and their asthma exacerbations lead to hospitalisations twice as often compared to patients with non‐NERD‐asthma. NERD patients are prone to recurrent nasal polyposis requiring frequent endoscopic sinus surgeries. However, the early risk factors of NERD are not fully understood. The aim was to identify risk factors of NERD among patients with adult‐onset asthma.

**Methods:**

We used data from 1350 population‐based adult asthmatics with adult‐onset asthma from Finnish national registers. NERD was defined as self‐reported wheeze or other typical respiratory symptoms after ingestion of NSAIDs. Thirty‐six covariates covering several domains (personal characteristics, life‐style, early life factors, asthma characteristics and multimorbidities) were selected based on literature and were studied in association with NERD using logistic regressions.

**Results:**

The study population included 153 (11.3%) asthmatics with NERD. Thirty‐six covariates were entered in univariate logistic regression analysis, in which 23 were associated with NERD (*p* < 0.05). These variables were entered in a multivariable logistic regression model in which allergic respiratory symptoms, female sex, osteoarthritis, difficult asthma, nasal polyps, second‐hand smoke exposure at home, having 3 or more older siblings and being overweight were significantly associated with asthma with NERD (*p* < 0.05). Overweight decreased the risk of NERD, other factors increased it.

**Conclusion:**

According to our study, risk factors of NERD in part are associated with female sex, BMI, exposure to tobacco smoke, allergy, orthopaedic disorders and infection history, and their early recognition might thus be important to manage the burden of NERD.

## 1 BACKGROUND

NSAID‐exacerbated respiratory disease (NERD) is a hypersensitivity condition characterised by NSAID‐intolerance, asthma, or chronic rhinosinusitis with or without nasal polyps. Most patients develop this hypersensitivity during the course of their asthma/chronic rhinosinusitis, but sometimes the allergic response to NSAIDs may occur before the onset of their upper airway disease.^1^

NERD patients develop a reaction to NSAIDs within 30–180 min. The reaction includes upper and/or lower symptoms, such as nasal congestion, rhinorrhea, wheezing, coughing and shortness of breath.^1^

The prevalence of NERD in adult asthma patients is 3%–21% depending on the diagnostic methods used,^1^, ^2^, ^3^, ^4^ and the prevalence among patients with severe asthma is even higher.^2^ In Finland, 1.4% of the adult population is affected by NERD.^5^ It is associated with severe adult‐onset asthma.

Among different asthma phenotypes, NERD represents a difficult‐to‐treat asthma.^1^ Most NERD patients have moderate to severe asthma though some might have a milder disease.^1^ NERD patients are more prone to severe allergic reactions and anaphylaxis than patients with non‐NERD‐asthma.^5^ In addition, asthma exacerbations lead to hospitalisations twice as often with NERD patients than with patients with non‐NERD asthma.^5^ This stresses the importance of investigating the factors leading to NERD. In addition, NERD patients are more prone to recurrent nasal polyposis, which requires frequent endoscopic sinus surgeries.^1^, ^6^

Light has already been shed on risk factors of NERD. According to Andersén et al., risk factors for NERD include older age, family history of allergic rhinitis (AR) or asthma, long‐term smoking and exposure to pollutants and second‐hand tobacco smoke.^5^ The risk factors of NERD also include such as female sex, overweight, farming environment in the childhood, airborne occupational exposure and visible mould at home.^5^ ^,^ ^7^

We have previously identified in the Adult Asthma in Finland cohort that NERD is a risk factor for severe adult‐onset asthma.^8^ Our further aim was to study early and late risk factors of NERD among asthmatics. We hypothesised that risk factors for NERD might in part be related to infection history or lifestyle and may be in part related to risk factors of severe asthma and/or to the literature of the risk factors of NERD.

## 2 METHODS

### 2.1 Study design

This is a cross‐sectional population‐based case‐control study of adult‐onset asthma with NERD in Finland. We analysed in 2022–2023 questionnaire‐based data of childhood and adulthood factors. The questionnaire was performed in 1996–1997.

### 2.2 Setting

Population‐based sample of adult‐onset asthma patients with and without NERD in Finland.

### 2.3 Study population

We used data from Adult Asthma in Finland, a population‐based matched case‐control study, which was performed in 1996–1997. The data consisted of information from 1350 asthma patients older than 30 years of age. One hundred and eighty‐two asthma patients were from Mini Finland Health Survey, a longitudinal population‐based survey, and 1168 asthma patients were a random sample of recently diagnosed asthmatics from the Finnish Drug Reimbursement register. In Finland, the reimbursement right needs to be granted by a certificate made by the patient's physician. This certificate holds background information, clinical exam results, lung function test results and findings and conclusions after an asthma treatment test period of 6 months. All the asthma patients were appropriately physician‐diagnosed. They had self‐reported onset of asthma symptoms and/or an asthma diagnosis after 15 years of age. The questionnaire included demographic as well as asthma‐specific questions. Among the asthma patient group, the response rate was 84.6%. The ethical committee of Tampere University Hospital gave consent to the study, and all participants gave written informed consent to the study.

### 2.4 Outcomes

Individuals with asthma NERD were asked which substances cause asthma symptoms and exacerbations. A responder was defined as a NERD patient if they chose aspirin or reported any NSAID or generally ‘pain relievers’ in the question ‘From the following list, choose all the factors that you have noticed to exacerbate or cause your asthma or to cause wheezing and difficulty in breathing’.

Of these 1350 adult‐onset asthmatics, 153 were reported to have had asthma exacerbations due to aspirin or NSAIDs and thus were defined as NERD patients.

### 2.5 Covariates

Thirty‐six covariates were a priori selected based on their potential impact on severe asthma from the data reported in the literature:

‐ Personal characteristics (3 covariates): sex,^5^ overweight (BMI >25),^5^ and underweight (BMI <20).
‐ Asthma/NERD characteristics (6 covariates): age as asthma onset (<40 or ≥40), self‐reported difficult asthma,^1^ nasal polyps,^1^ use of oral corticosteroids regularly or in courses,^1^ waking up to asthma symptoms at night several times a month,^1^ daily use of inhaled corticosteroids and short‐acting beta agonists (SABA)^1^ (ICS as a continuous daily medication and SABA occasionally as a symptom reliever).
‐ Smoking status (3 covariates)^5^: Has the patient ever been a smoker? Passive smoking status: Is the patient currently exposed to second‐hand tobacco smoke by living with a person who smokes indoors at home? Did the patient's parents smoke during the patient's childhood?
‐ Childhood in a farming environment (2 covariates)^5^: childhood on a farm and childhood in a rural area.
‐ Sibling characteristics (1 covariate): position among siblings (1. child/2.–3. child/4. or later child).
‐ History of different respiratory infections (12 covariates): Has the patient had frequent respiratory infections before school, frequent respiratory infections in school, frequent respiratory infections in adulthood, has the patient ever had pneumonia, has the patient had severe childhood infections (pneumonia, hospitalised due to an infection/contagious disease), number of physician‐diagnosed bronchitis in the last year (0/1–2/3 or over), number of physician‐diagnosed otitis in the last year (0/1 or over), number of physician‐diagnosed pneumonia in the last year (0/1 or over), number of physician‐diagnosed sinusitis in the last year (0/1–2/3 or over), number of flus with a fever in the last year (0/1–3/4 or over), number of physician‐diagnosed angina in the last year (0/1 or over), has the patient been unable to work for ≥21 days due to respiratory infection(s) in the last year.
‐ Allergy characteristics (5 covariates): does the patient have AR,^5^ atopic dermatitis, allergic conjunctivitis (AC), have the patient ever had allergic respiratory symptoms, do patient's parents have asthma or allergy.^9^
‐ Other diseases (4 covariates): osteoarthritis, musculoskeletal back disease, rheumatism, use of hypertension medication.

Missing answers were included in the study and regarded as ‘no’, except in three variables, sex, overweight and underweight, in which they were regarded as ‘no answer’.

### 2.6 Statistical analyses

The associations between each risk factor and NERD were estimated using chi‐square (dichotomous) and *t*‐test (continuous), and by using univariate logistic regressions. Odds ratios (OR) with 95% confidence intervals (CIs) are reported. Risk factors associated with NERD with a *p*‐value below 0.05 were included in a multivariable logistic regression model. Statistical analyses were carried out using R 4.1.0,^10^ RStudio,^11^ dplyr^12^ and the stats^10^ packages. Variables that had three different possible values were made categorical before the univariate and multivariable analyses.

Based on significant changes in OR values between univariate and multivariable analyses, we suspected that ‘Allergic respiratory symptoms’ and ‘Female sex’ could act as effect modifiers in the multivariable analysis. Thus, we used multivariable regression with interaction variables to evaluate this. We made interaction variables by coupling these variables with other variables selected based on logical associations and literature, and entered these interaction variables in separate multivariable analyses. The multivariable analyses were similar to the original multivariable analysis, but included one interaction variable at a time. ‘Allergic respiratory symptoms’ was coupled with nine other variables (‘Self‐reported difficult asthma’, ‘Ever smoking’, ‘Second‐hand smoke exposure at home’, ‘Atopic dermatitis’, ‘Allergic rhinitis’, ‘Allergic rhinoconjunctivitis’, ‘Oral corticosteroids regularly or in courses’, ‘Waking up to symptoms at night several times a month’, ‘Unable to work for 21 days or over in the last year due to respiratory infection(s)’) and ‘Female sex’ was coupled with four other variables (‘Self‐reported difficult asthma’, ‘Second‐hand smoke exposure at home’, ‘Allergic respiratory symptoms’, ‘Osteoarthritis’). After this, we performed new multivariable analyses in which we excluded interacting variables one at a time from the original multivariable analysis, in order to see if there was a change in the resulting significant variables.

We studied which variables are associated with NERD in two age groups to investigate the effect of changes in living environment and lifestyle during different decades. Group 1 included subjects born in 1943–1966 and group 2 those born in 1904–1942.

We investigated which variables are associated with uncontrolled asthma compared with controlled asthma. This comparison was performed in two groups separately: those having asthma with NERD (NERD‐group) and those having asthma without NERD (non‐NERD group). Uncontrolled asthma was defined as reporting wake‐ups to asthma symptoms at night several times a month and/or daily use of ICS and SABA.

## 3 RESULTS

### 3.1 Population description

**FIGURE 1:**
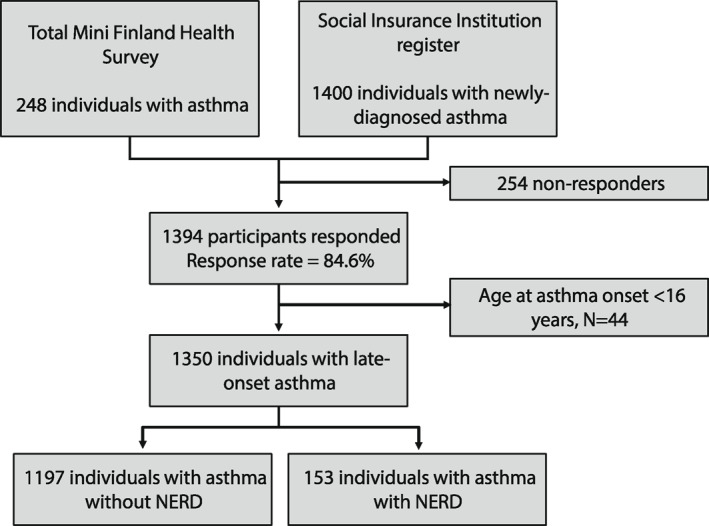
Flowchart of the study.

The study flow chart is shown in Figure 1. The total number of adult‐onset asthma cases with available data was 1350. Mean age (SD, min–max) was 54.39 (12.24, 31–93) years. The proportion of females was 62.1%, and the proportion of subjects reporting at least secondary school level of education was 36.1%. A hundred and fifty‐three (11.3%) (153 responders) of the study population reported having NERD, which is in line with the previous findings that 3%–21% of adult asthma patients have NERD.^1^, ^2^, ^3^, ^4^ The data dates back to 1997 and includes responders born in the early 1900s (mean = 1942.6, SD = 12.2, min–max = 1904–1966). The mean age (SD, min–max) among the study population was 54.39 years (12.24, 31–93) and among the NERD patients 54.15 years (12.24, 31–93). 37.9% of the study population were male, 18.3% of the NERD patients were male. 11.2% of the study population reported having severe asthma, and 19.0% of the NERD patients reported having severe asthma.

### 3.2 Risk factors of NERD

The description of self‐reported demographic factors in the asthma with NERD and asthma without NERD groups is shown in Table 1. In unadjusted analysis, asthma with NERD was statistically significantly associated (*p* < 0.05) with female sex, overweight, allergic respiratory symptoms, several physician‐diagnosed sinusitis in the last year, several physician‐diagnosed bronchitis in the last year, nasal polyps, recurrent respiratory infections before school age, oral corticosteroids regularly or in courses, feverish flu several times in the last year, waking up to symptoms at night several times a month, recurrent respiratory infections in adulthood, unable to work for 21 days or over in the last year due to respiratory infection(s), self‐reported difficult asthma, ever smoking, second‐hand smoke exposure at home, having ever had pneumonia, musculoskeletal back disease, rheumatoid arthritis, having 3 or more older siblings, allergic dermatitis (AD), AR, allergic rhinoconjunctivitis and osteoarthritis (Table 2).

**TABLE 1:** Description of the study population.

	Non‐NERD	NERD	Total response count (non‐NERD)^a^	Total response count (NERD)^a^
	*N* (% of non‐NERD total response count)	*N* (% of NERD total response count)		
Sex				
Male	484 (40.4)	28 (18.3)	1197	153
Female	713 (59.6)	125 (81.7)		
Underweight (BMI <20)				
No	1110 (95.4)	143 (94.7)	1164	151
Yes	54 (4.6)	8 (5.3)		
NA	33	2		
Overweight (BMI >25)				
No	406 (34.9)	71 (47.0)	1164	151
Yes	758 (65.1)	80 (53.0)		
NA	33	2		
Age as asthma onset 40 or over				
No	293 (24.5)	43 (28.1)	1197	153
Yes	904 (75.5)	110 (71.9)		
Self‐reported difficult asthma				
No	1075 (89.8)	124 (81.0)	1197	153
Yes	122 (10.2)	29 (19.0)		
Oral corticosteroid use regularly or in courses				
No	857 (71.6)	95 (62.1)	1197	153
Yes	340 (28.4)	58 (37.9)		
Waking up at night to asthma symptoms several times a month				
No	787 (65.7)	85 (55.6)	1197	153
Yes	410 (34.3)	68 (44.4)		
ICS and SABA daily				
No	956 (79.9)	118 (77.1)	1197	153
Yes	241 (20.1)	35 (22.9)		
Nasal polyps				
No	1072 (89.6)	126 (82.4)	1197	153
Yes	125 (10.4)	27 (17.6)		
Allergic rhinitis				
No	613 (51.2)	52 (34.0)	1197	153
Yes	584 (48.8)	101 (66.0)		
Atopic dermatitis				
No	815 (68.1)	78 (51.0)	1197	153
Yes	382 (31.9)	75 (49.0)		
Allergic rhinoconjunctivitis				
No	737 (61.6)	64 (41.8)	1197	153
Yes	460 (38.4)	89 (58.2)		
Allergic respiratory symptoms				
No	602 (50.3)	38 (24.8)	1197	153
Yes	595 (49.7)	115 (75.2)		
At least one parent with asthma				
No	790 (66.0)	94 (61.4)	1197	153
Yes	407 (34.0)	59 (38.6)		
Osteoarthritis				
No	1000 (83.5)	106 (69.3)	1197	153
Yes	197 (16.5)	47 (30.7)		
Back disease				
No	884 (73.9)	99 (64.7)	1197	153
Yes	313 (26.1)	54 (35.3)		
Rheumatoid arthritis				
No	1149 (96.0)	141 (92.2)	1197	153
Yes	48 (4.0)	12 (7.8)		
Hypertension medication				
No	936 (78.2)	116 (75.8)	1197	153
Yes	261 (21.8)	37 (24.2)		
Recurrent respiratory infections before school age				
No	1127 (94.2)	133 (86.9)	1197	153
Yes	70 (5.8)	20 (13.1)		
Recurrent respiratory infections in school age				
No	971 (81.1)	117 (76.5)	1197	153
Yes	226 (18.9)	36 (23.5)		
Recurrent respiratory infections in adulthood				
No	497 (41.5)	46 (30.1)	1197	153
Yes	700 (58.5)	107 (69.9)		
Severe infections in childhood				
No	999 (83.5)	123 (80.4)	1197	153
Yes	198 (16.5)	30 (19.6)		
Pneumonia ever				
No	851 (71.1)	94 (61.4)	1197	153
Yes	346 (28.9)	59 (38.6)		
Physician‐diagnosed pneumonia in the last year				
No	1170 (97.7)	149 (97.4)	1197	153
Yes	27 (2.3)	4 (2.6)		
Physician‐diagnosed otitis in the last year				
No	1144 (95.6)	144 (94.1)	1197	153
Yes	53 (4.4)	9 (5.9)		
Physician‐diagnosed angina in the last year				
No	1166 (97.4)	152 (99.3)	1197	153
Yes	31 (2.6)	1 (0.7)		
Physician‐diagnosed sinusitis in the last year				
0/unknown	1035 (86.5)	117 (76.5)	1197	153
1–2	137 (11.4)	26 (17.0)		
3+	25 (2.1)	10 (6.5)		
Physician‐diagnosed bronchitis in the last year				
0/unknown	954 (79.7)	104 (68.0)	1197	153
1–2	203 (17.0)	44 (28.8)		
3+	40 (3.3)	5 (3.3)		
Feverish flu in the last year				
0/unknown	845 (70.6)	90 (58.8)	1197	153
1–3	327 (27.3)	57 (37.3)		
4+	25 (2.1)	6 (3.9)		
Unable to work for 21 days or over in the past year due to respiratory infection(s)				
No	1092 (91.2)	130 (85.0)	1197	153
Yes	105 (8.8)	23 (15.0)		
Smoking ever				
No	481 (40.2)	75 (49.0)	1197	153
Yes	716 (59.8)	78 (51.0)		
Smoker parent(s)				
No	574 (48.0)	75 (49.0)	1197	153
Yes	623 (52.0)	78 (51.0)		
Second‐hand tobacco smoke exposure at home				
No	1145 (95.7)	140 (91.5)	1197	153
Yes	52 (4.3)	13 (8.5)		
4. or later child				
No	862 (72.0)	93 (60.8)	1197	153
Yes	335 (28.0)	60 (39.2)		
Childhood spent on a farm				
No	734 (61.3)	84 (54.9)	1197	153
Yes	463 (38.7)	69 (45.1)		
Childhood spent on countryside				
No	303 (25.3)	40 (26.1)	1197	153
Yes	894 (74.7)	113 (73.9)		

*Note*: Missing answers were included and regarded as ‘no’, except in the variables ‘sex’, ‘overweight’ and ‘underweight’, where they were regarded as ‘no answer’.

Abbreviations: ICS, inhaled corticosteroids; *N*, number; NERD, NSAID exacerbated respiratory disease; SABA, short‐acting beta agonists.

^a^ NA values are not included in the total response count.

**TABLE 2:** Results from the univariate and multivariable regression models.

	Model 1	Model 1	Model 2	Model 2
	Univariate regression model	Univariate regression model	Multivariable regression model	Multivariable regression model
	OR1 (95% CI)	*p*1	OR2 (95% CI)	*p*2
Sex				
Male	1		1	
Female	3.03 (1.98–4.64)	**<0.001**	2.50 (1.52–4.09)	**<0.001**
Underweight (BMI <20)				
No	1			
Yes	1.15 (0.54–2.47)	0.72	Not entered	
Overweight (BMI >25)				
No	1		1	
Yes	0.6 (0.43–0.85)	**0.0038**	0.65 (0.45–0.94)	**0.023**
Age as asthma onset 40 or over				
No	1			
Yes	0.83 (0.57–1.21)	0.33	Not entered	
Self‐reported difficult asthma				
No	1		1	
Yes	2.06 (1.32–3.22)	**0.0015**	2.21 (1.28–3.82)	**0.0045**
Oral corticosteroid use regularly or in courses				
No	1		1	
Yes	1.54 (1.08–2.18)	**0.016**	1.16 (0.77–1.75)	0.48
Waking up at night to asthma symptoms several times a month				
No	1		1	
Yes	1.54 (1.09–2.16)	**0.014**	1.19 (0.80–1.79)	0.39
ICS and SABA daily				
No	1			
Yes	1.18 (0.79–1.76)	0.43	Not entered	
Nasal polyps				
No	1		1	
Yes	1.84 (1.17–2.90)	**0.0088**	1.76 (1.07–2.90)	**0.026**
Allergic rhinitis				
No	1		1	
Yes	2.04 (1.43–2.90)	**<0.001**	1.06 (0.69–1.62)	0.80
Atopic dermatitis				
No	1		1	
Yes	2.05 (1.46–2.88)	**<0.001**	1.26 (0.85–1.87)	0.25
Allergic rhinoconjunctivitis				
No	1		1	
Yes	2.23 (1.58–3.14)	**<0.001**	1.23 (0.81–1.88)	0.34
Allergic respiratory symptoms				
No	1		1	
Yes	3.06 (2.09–4.49)	**<0.001**	1.91 (1.21–3.02)	**0.0055**
At least one parent with asthma				
No	1			
Yes	1.22 (0.86–1.72)	0.26	Not entered	
Osteoarthritis				
No	1		1	
Yes	2.25 (1.55–3.28)	**<0.001**	1.90 (1.23–2.95)	**0.0042**
Back disease				
No	1		1	
Yes	1.54 (1.08–2.20)	**0.017**	1.02 (0.67–1.54)	0.94
Rheumatoid arthritis				
No	1		1	
Yes	2.04 (1.06–3.93)	**0.034**	1.49 (0.71–3.13)	0.30
Hypertension medication				
No	1			
Yes	1.14 (0.77–1.70)	0.50	Not entered	
Recurrent respiratory infections before school age				
No	1		1	
Yes	2.42 (1.43–4.11)	**0.0010**	1.68 (0.93–3.06)	0.088
Recurrent respiratory infections in school age				
No	1			
Yes	1.32 (0.89–1.97)	0.17	Not entered	
Recurrent respiratory infections in adulthood				
No	1		1	
Yes	1.65 (1.15–2.38)	**0.0069**	0.96 (0.63–1.46)	0.84
Severe infections in childhood				
No	1			
Yes	1.23 (0.80–1.89)	0.34	Not entered	
Pneumonia ever				
No	1		1	
Yes	1.54 (1.09–2.19)	**0.015**	1.21 (0.81–1.80)	0.35
Physician‐diagnosed pneumonia in the last year				
No	1			
Yes	1.16 (0.40–3.37)	0.78	Not entered	
Physician‐diagnosed otitis in the last year				
No	1			
Yes	1.35 (0.65–2.79)	0.42	Not entered	
Physician‐diagnosed angina in the last year				
No	1			
Yes	0.25 (0.03–1.82)	0.17	Not entered	
Physician‐diagnosed sinusitis in the last year				
0/unknown	1			
1–2	1.68 (1.06–2.66)	**0.028**	1.05 (0.62–1.78)	0.84
3+	3.54 (1.66–7.55)	**0.0011**	1.95 (0.82–4.64)	0.13
Physician‐diagnosed bronchitis in the last year				
0/unknown	1			
1–2	1.99 (1.35–2.92)	**<0.001**	1.40 (0.90–2.19)	0.14
3+	1.15 (0.44–2.97)	0.78	0.50 (0.17–1.49)	0.21
Feverish flu in the last year				
0/unknown	1			
1–3	1.64 (1.15–2.34)	**0.0066**	1.43 (0.96–2.12)	0.078
4+	2.25 (0.90–5.64)	0.083	1.36 (0.49–3.76)	0.55
Unable to work for 21 days or over in the past year due to respiratory infection(s)				
No	1		1	
Yes	1.84 (1.13–2.99)	**0.014**	1.35 (0.77–2.35)	0.29
Smoking ever				
No	1		1	
Yes	0.70 (0.50–0.98)	**0.037**	0.90 (0.61–1.33)	0.60
Smoker parent(s)				
No	1			
Yes	0.96 (0.68–1.34)	0.80	Not entered	
Second‐hand tobacco smoke exposure at home				
No	1		1	
Yes	2.04 (1.09–3.85)	**0.027**	2.38 (1.18–4.79)	**0.016**
4. or later child				
No	1		1	
Yes	1.66 (1.17–2.35)	**0.0043**	1.63 (1.10–2.41)	**0.014**
Childhood spent on a farm				
No	1			
Yes	1.30 (0.93–1.83)	0.13	Not entered	
Childhood spent on countryside				
No	1			
Yes	0.96 (0.65–1.40)	0.82	Not entered	

*Note*: Model 1 = Univariate analysis. Model 2 = Multivariable analysis by the 23 variables that were associated at *p* < 0.05 level in the Model 1. *p* < 0.05 is considered significant. As described in methods, interaction models were added individually to the multivariable model. The resulting *p*‐values for the interaction variables were allergic respiratory symptoms × self‐reported difficult asthma = 0.28, allergic respiratory symptoms × allergic rhinoconjunctivitis = 0.19, allergic respiratory symptoms × atopic dermatitis = 0.23, allergic respiratory symptoms × waking up to asthma symptoms at night several times a month = 0.18, allergic respiratory symptoms × smoking ever = 0.51, allergic respiratory symptoms × second‐hand smoke exposure at home = 0.96, allergic respiratory symptoms × allergic rhinitis = 0.59, allergic respiratory symptoms × oral corticosteroid use regularly or in courses = 0.47, allergic respiratory symptoms × unable to work for 21 days or over in the past year due to respiratory infection(s) = 0.66, female sex × second‐hand smoke exposure at home = 0.20, female sex × self‐reported difficult asthma = 0.11, female sex × osteoarthritis = 0.90, female sex × allergic respiratory symptoms = 0.40.

Abbreviations: CI, confidence interval; ICS, inhaled corticosteroids; OR, odds ratio; *p*, *p*‐value; SABA, short‐acting beta agonists.

The variables that were associated with NERD at level *p* < 0.05 were entered in a multivariable regression model (Table 2). In the multivariable model, asthma with NERD was significantly associated with female sex (OR [CI 95%] = 2.50 [1.52–4.09]), overweight (0.65 [0.45–0.94]), allergic respiratory symptoms (1.91 [1.21–3.02]), self‐reported difficult asthma (2.21 [1.28–3.82]), nasal polyps (1.76 [1.07–2.90]), second‐hand tobacco smoke exposure at home (2.38 [1.18–4.79]), having 3 or more older siblings (1.63 [1.10–2.41]), and osteoarthritis (1.90 [1.23–2.95]), when compared to the asthma without NERD‐phenotype.

We finally conducted further analysis by adding interaction terms to the multivariable analysis. Six of the interaction variables were significant at level *p* < 0.3: ‘Allergic respiratory symptoms & Self‐reported difficult asthma’ (adjusted *p*‐value 0.28, Table 2), ‘Allergic respiratory symptoms & Allergic rhinoconjunctivitis’ (adjusted *p*‐value 0.19, Table 2), ‘Allergic respiratory symptoms & Atopic dermatitis’ (adjusted *p*‐value 0.23 Table 2), ‘Allergic respiratory symptoms & Waking up to symptoms at night several times a month’ (adjusted *p*‐value 0.18, Table 2), ‘Female sex & Second‐hand smoke exposure at home’ (adjusted *p*‐value 0.20, Table 2) and ‘Female sex & Self‐reported difficult asthma’ (adjusted *p*‐value 0.11, Table 2). When we excluded interacting variables one at a time from the original multivariable analysis, the significant variables stayed the same apart from excluded variables compared to the original multivariable analysis.

### 3.3 Risk factors of NERD in the two age groups

We studied which variables are associated with NERD in two age groups by logistic regression. Group 1 included younger asthmatics born in 1943–1966 (total *N* = 667, NERD patients *N* = 80). The age of the participants ranged between 31 and 54 years. In this younger group 1, self‐reported difficult asthma and nasal polyps were significantly associated with NERD in the multivariable analysis (Table 3). There was an insignificant trend that high BMI was associated with NERD in the younger group (Table 3), however decreasing the risk of NERD. Group 2 included individuals who were born in 1904–1942 (total *N* = 683, NERD patients *N* = 73). The age of the participants was ranging between 55 and 93 years. In this older group 2, female sex, allergic respiratory symptoms and osteoarthritis were significantly associated with NERD in the multivariable analysis (Table 4).

**TABLE 3:** Results from age‐stratified analysis for group 1 (patients born between 1943 and 1966).

	Univariate regression model	Univariate regression model	Multivariable regression model	Multivariable regression model
	OR1 (95% CI)	*p*1	OR2 (95% CI)	*p*2
Sex				
Male	1		1	
Female	1.89 (1.07–3.31)	**0.027**	1.70 (0.90–3.21)	0.10
Underweight (BMI <20)				
No	1			
Yes	1.45 (0.62–3.37)	0.39	Not entered	
Overweight (BMI >25)				
No	1		1	
Yes	0.61 (0.38–0.98)	**0.041**	0.65 (0.39–1.08)	0.096
Age as asthma onset 40 or over				
No	1			
Yes	0.83 (0.52–1.32)	0.43	Not entered	
Self‐reported difficult asthma				
No	1		1	
Yes	2.65 (1.40–5.01)	**0.0026**	3.11 (1.47–6.57)	**0.0030**
Oral corticosteroid use regularly or in courses				
No	1		1	
Yes	1.72 (1.06–2.79)	**0.028**	1.31 (0.76–2.26)	0.33
Waking up at night to asthma symptoms several times a month				
No	1			
Yes	1.25 (0.76–2.05)	0.38	Not entered	
ICS and SABA daily				
No	1			
Yes	1.12 (0.58–2.16)	0.74	Not entered	
Nasal polyps				
No	1		1	
Yes	2.20 (1.19–4.04)	**0.011**	1.99 (1.03–3.84)	**0.041**
Allergic rhinitis				
No	1			
Yes	1.60 (0.97–2.62)	0.065	Not entered	
Atopic dermatitis				
No	1		1	
Yes	2.44 (1.51–3.96)	**<0.001**	1.60 (0.93–2.75)	0.092
Allergic rhinoconjunctivitis				
No	1		1	
Yes	1.93 (1.19–3.13)	**0.0078**	1.13 (0.62–2.04)	0.69
Allergic respiratory symptoms				
No	1		1	
Yes	2.43 (1.44–4.1)	**<0.001**	1.75 (0.95–3.23)	0.073
At least one parent with asthma				
No	1			
Yes	1.06 (0.67–1.70)	0.80	Not entered	
Osteoarthritis				
No	1			
Yes	1.97 (0.97–3.99)	0.061	Not entered	
Back disease				
No	1		1	
Yes	2.00 (1.20–3.33)	**0.0079**	1.55 (0.88–2.72)	0.13
Rheumatoid arthritis				
No	1			
Yes	3.04 (0.93–9.92)	0.066	Not entered	
Hypertension medication				
No	1			
Yes	1.54 (0.82–2.89)	0.18	Not entered	
Recurrent respiratory infections before school age				
No	1		1	
Yes	2.18 (1.12–4.23)	**0.021**	1.65 (0.79–3.42)	0.18
Recurrent respiratory infections in school age				
No	1			
Yes	1.19 (0.71–1.99)	0.52	Not entered	
Recurrent respiratory infections in adulthood				
No	1			
Yes	1.25 (0.76–2.06)	0.37	Not entered	
Severe infections in childhood				
No	1			
Yes	1.40 (0.79–2.46)	0.25	Not entered	
Pneumonia ever				
No	1		1	
Yes	1.94 (1.20–3.15)	**0.0072**	1.69 (0.99–2.89)	0.053
Physician‐diagnosed pneumonia in the last year				
No	1			
Yes	2.12 (0.43–10.41)	0.35	Not entered	
Physician‐diagnosed otitis in the last year				
No	1			
Yes	1.44 (0.65–3.19)	0.37	Not entered	
Physician‐diagnosed angina in the last year				
No	1			
Yes	0 (0‐∞)	0.98	Not entered	
Physician‐diagnosed sinusitis in the last year				
0/unknown	1		1	
1–2	1.67 (0.92–3.06)	0.094	1.15 (0.58–2.28)	0.69
3+	3.95 (1.72–9.11)	**0.0013**	2.54 (0.96–6.71)	0.060
Physician‐diagnosed bronchitis in the last year				
0/unknown	1		1	
1–2	1.89 (1.11–3.21)	**0.020**	1.24 (0.68–2.28)	0.48
3+	0.90 (0.27–3.08)	0.87	0.32 (0.08–1.31)	0.11
Feverish flu in the last year				
0/unknown	1		1	
1–3	1.88 (1.16–3.04)	**0.010**	1.49 (0.88–2.53)	0.14
4+	2.30 (0.74–7.20)	0.15	1.47 (0.41–5.31)	0.55
Unable to work for 21 days or over in the past year due to respiratory infection(s)				
No	1		1	
Yes	1.93 (1.02–3.66)	**0.042**	1.20 (0.58–2.50)	0.62
Smoking ever				
No	1			
Yes	0.91 (0.56–1.47)	0.70	Not entered	
Smoker parent(s)				
No	1			
Yes	1.16 (0.72–1.85)	0.54	Not entered	
Second‐hand tobacco smoke exposure at home				
No	1			
Yes	2.22 (0.97–5.05)	0.058	Not entered	
4. or later child				
No	1			
Yes	1.52 (0.92–2.52)	0.10	Not entered	
Childhood spent on a farm				
No	1			
Yes	1.35 (0.83–2.19)	0.23	Not entered	
Childhood spent on countryside				
No	1			
Yes	0.92 (0.56–1.50)	0.73	Not entered	

*Note*: *p* < 0.05 is considered significant. Patients with asthma with NERD *N* = 80 (12.0%), patients with non‐NERD asthma *N* = 587 (88.0%).

Abbreviations: CI, confidence interval; ICS, inhaled corticosteroids; NERD, NSAID‐exacerbated respiratory disease; OR, odds ratio; *p*, *p*‐value; SABA, short‐acting beta agonists.

**TABLE 4:** Results from age‐stratified analysis of group 2 (patients born between 1904 and 1942).

	Univariate regression model	Univariate regression model	Multivariable regression model	Multivariable regression model
	OR1 (95% CI)	*p*1	OR2 (95% CI)	*p*2
Sex				
Male	1		1	
Female	4.98 (2.57–9.63)	**<0.001**	4.29 (1.99–9.25)	**<0.001**
Underweight (BMI <20)				
No	1			
Yes	0.45 (0.060–3.42)	0.44	Not entered	
Overweight (BMI >25)				
No	1			
Yes	0.60 (0.36–1.01)	0.053	Not entered	
Age as asthma onset 40 or over				
No	1			
Yes	0.95 (0.42–2.18)	0.91	Not entered	
Self‐reported difficult asthma				
No	1			
Yes	1.69 (0.90–3.18)	0.10	Not entered	
Oral corticosteroid use regularly or in courses				
No	1			
Yes	1.36 (0.82–2.27)	0.23	Not entered	
Waking up at night to asthma symptoms several times a month				
No	1		1	
Yes	1.98 (1.21–3.22)	**0.0065**	1.65 (0.96–2.83)	0.071
ICS and SABA daily				
No	1			
Yes	1.28 (0.76–2.17)	0.35	Not entered	
Nasal polyps				
No	1			
Yes	1.49 (0.75–2.97)	0.26	Not entered	
Allergic rhinitis				
No	1		1	
Yes	2.57 (1.55–4.26)	**<0.001**	1.34 (0.74–2.40)	0.33
Atopic dermatitis				
No	1		1	
Yes	1.69 (1.00–2.83)	**0.048**	0.81 (0.45–1.46)	0.48
Allergic rhinoconjunctivitis				
No	1		1	
Yes	2.59 (1.59–4.24)	**<0.001**	1.16 (0.64–2.08)	0.63
Allergic respiratory symptoms				
No	1		1	
Yes	3.88 (2.21–6.83)	**<0.001**	2.22 (1.14–4.30)	**0.018**
At least one parent with asthma				
No	1			
Yes	1.39 (0.81–2.40)	0.23	Not entered	
Osteoarthritis				
No	1		1	
Yes	2.91 (1.77–4.76)	**<0.001**	2.04 (1.20–3.47)	**0.0081**
Back disease				
No	1			
Yes	1.29 (0.78–2.12)	0.33	Not entered	
Rheumatoid arthritis				
No	1			
Yes	1.85 (0.83–4.14)	0.13	Not entered	
Hypertension medication				
No	1			
Yes	1.02 (0.60–1.71)	0.95	Not entered	
Recurrent respiratory infections before school age				
No	1		1	
Yes	2.83 (1.17–6.89)	**0.021**	1.57 (0.57–4.27)	0.38
Recurrent respiratory infections in school age				
No	1			
Yes	1.50 (0.79–2.86)	0.22	Not entered	
Recurrent respiratory infections in adulthood				
No	1		1	
Yes	2.19 (1.28–3.75)	**0.0043**	1.39 (0.77–2.50)	0.28
Severe infections in childhood				
No	1			
Yes	1.04 (0.54–2.00)	0.91	Not entered	
Pneumonia ever				
No	1			
Yes	1.24 (0.75–2.05)	0.40	Not entered	
Physician‐diagnosed pneumonia in the last year				
No	1			
Yes	0.83 (0.19–3.63)	0.81	Not entered	
Physician‐diagnosed otitis in the last year				
No	1			
Yes	0.76 (0.10–5.94)	0.79	Not entered	
Physician‐diagnosed angina in the last year				
No	1			
Yes	0.59 (0.08–4.56)	0.61	Not entered	
Physician‐diagnosed sinusitis in the last year				
0/unknown	1			
1–2	1.68 (0.81–3.47)	0.16		
3+	1.78 (0.20–15.49)	0.60	Not entered	
Physician‐diagnosed bronchitis in the last year				
0/unknown	1		1	
1–2	2.09 (1.20–3.64)	**0.0089**	1.57 (0.85–2.90)	0.15
3+	1.66 (0.36–7.63)	0.51	1.24 (0.24–6.29)	0.79
Feverish flu in the last year				
0/unknown	1			
1–3	1.34 (0.77–2.35)	0.31		
4+	2.27 (0.47–10.99)	0.31	Not entered	
Unable to work for 21 days or over in the past year due to respiratory infection(s)				
No	1			
Yes	1.68 (0.79–3.60)	0.18	Not entered	
Smoking ever				
No	1		1	
Yes	0.51 (0.31–0.83)	**0.0076**	1.12 (0.62–2.02)	0.71
Smoker parent(s)				
No	1			
Yes	0.78 (0.48–1.27)	0.31	Not entered	
Second‐hand tobacco smoke exposure at home				
No	1			
Yes	1.80 (0.66–4.86)	0.25	Not entered	
4. or later child				
No	1		1	
Yes	1.87 (1.14–3.05)	**0.012**	1.69 (0.99–2.87)	0.054
Childhood spent on a farm				
No	1			
Yes	1.33 (0.82–2.17)	0.25	Not entered	
Childhood spent on countryside				
No	1			
Yes	1.11 (0.58–2.12)	0.76	Not entered	

*Note*: *p* < 0.05 is considered significant. Patients with asthma with NERD *N* = 73 (10.7%), patients with non‐NERD asthma *N* = 610 (89.3%).

Abbreviations: CI, confidence interval; ICS, inhaled corticosteroids; NERD, NSAID‐exacerbated respiratory disease; OR, odds ratio; *p*, *p*‐value; SABA, short‐acting beta agonists.

### 3.4 Risk factors of uncontrolled asthma in the NERD and non‐NERD ‐groups

We investigated which variables are associated with uncontrolled asthma compared with controlled asthma. Uncontrolled asthma was defined as wake‐ups at night due to asthma symptoms several times a month (NERD group *N* = 44, non‐NERD group *N* = 287), daily ICS and SABA use (NERD group *N* = 11, non‐NERD group *N* = 118), or both (NERD group *N* = 24, non‐NERD group *N* = 123).

The risk factors of uncontrolled asthma were analysed in the two subgroups, those having asthma with NERD (NERD‐group *N* = 153) and those having asthma without NERD (non‐NERD group *N* = 1197). The results are in Tables S1 and S2.

In the NERD‐group, uncontrolled asthma was significantly associated with allergic respiratory symptoms and self‐reported difficult asthma in the multivariable analysis (Table S1). In the non‐NERD group, uncontrolled asthma was significantly associated with the following variables in the multivariable analysis: male, age at onset of asthma 40 or over, allergic respiratory symptoms, several physician‐diagnosed bronchitis in the last year, self‐reported difficult asthma, oral corticosteroid use regularly or in courses, recurrent respiratory infections in adulthood, back disease, physician‐diagnosed pneumonia in the last year (Table S2).

## 4 DISCUSSION

In this population‐based case‐control study of adult‐onset asthma, NERD was associated in the adjusted analysis with female sex, overweight, allergic respiratory symptoms, self‐reported difficult asthma, nasal polyps, second‐hand smoke exposure at home, having 3 or more older siblings, and osteoarthritis. All other associated factors increased the risk of NERD, but overweight decreased it, when looking at the OR value. Some of these factors (such as nasal polyps,^1^ female sex,^4^, ^5^, ^6^ BMI,^5^, ^7^ exposure to tobacco smoke^5^, ^7^) have been reported earlier, whereas a few of them (such as osteoarthritis, having many older siblings) seem to be novel.

Variables that showed a significant association with NERD in the unadjusted analysis, yet not in the adjusted analysis, were several physician‐diagnosed sinusitis in the last year, several physician‐diagnosed bronchitis in the last year, feverish flu(s) in the last year, recurrent respiratory infections before school age, oral corticosteroids regularly or in courses, waking up to symptoms at night several times a month, recurrent respiratory infections in adulthood, unable to work for 21 days or over the last year due to respiratory infection(s), ever smoking, having ever had pneumonia, musculoskeletal back disease, rheumatoid arthritis, AD, AR and allergic rhinoconjunctivitis.

In our study population, the prevalence of NERD was 11.3% in patients with adult‐onset asthma. This is in line with previous observations,^1^ in which the prevalence of NERD in adult asthmatics has been estimated to be 3%–21% depending on the diagnostic methods.^1^, ^2^, ^3^, ^4^

Previous studies of these data have focused on risk factors of asthma,^13^, ^14^ asthma mortality,^15^ or severe asthma,^8^ whereas risk factors of NERD have not previously been studied from these data.

In this current study, the main analysis showed that females are more likely to be affected by NERD, which was noted by Andersén et al.^5^ in their cross‐sectional population‐based study of 7930 adult participants from Finland, as well as in other articles.^4^, ^6^ Previous articles have declared that adult females are more at risk than adult males for developing asthma in general as well as severe asthma and later onset.^16^, ^17^ According to previous articles, asthma prevalence is higher in males prior to puberty, but after puberty, women are more at risk for developing asthma.^18^ This is in line with our finding, since NERD is more of an adult‐onset than a child‐onset type of asthma, with its first symptoms appearing around 30 years of age.^4^, ^6^ On the contrary, our group recently observed male sex to be a risk factor for severe adult‐onset asthma using the same data as in this study.

To investigate the effect of changes in living environment and lifestyle during different decades, we studied the risk factors of NERD in two age groups. Both groups showed an association between NERD and factors related to respiratory symptoms: asthma difficulty in the younger group and allergic respiratory symptoms in the older group. The younger group had nasal polyps as one of the associated factors, whereas the older group did not. The younger group showed an insignificant trend of association between NERD and variables related to infection history, whereas the older group had an insignificant association between NERD and three or more older siblings. The number of siblings in the older group might be reflecting their childhood infection burden; thus, both of these groups might be showing some association with NERD in infection‐related variables. Only the older group had osteoarthritis as a NERD‐associated factor, which might be explained by its higher prevalence among ageing individuals. In the older group, female sex was significantly associated with NERD. There was a similar, although insignificant, trend in the younger group.

In the younger group there was an insignificant trend that overweight is associated with asthma with NERD, however decreasing the risk of NERD. This trend was not seen in the older age group. Overweight also decreased the odds of having NERD in the whole population of our study. The results might reflect the increase in obesity and overweight during the last few decades in Finland,^19^ as well as that obesity might play a minor role in the aetiopathogenesis of NERD,^20^ and be associated with asthma also independently of NERD among adults.^20^ Previous studies of other study groups have shown that obesity increases the odds of a more persistent and severe asthma phenotype,^21^ and that obesity‐associated severe asthma may represent a distinct clinical phenotype.^22^ Interestingly, BMI was shown to be associated with NERD in the study by Andersén et al.,^5^ which observed that the majority of NERD patients were female and overweight. A population‐based study of 18,087 adult participants from West Sweden by Eriksson et al.^7^ found BMI to be a strong risk factor for NERD with a dose‐response relationship. Taken together, more studies on the role of BMI as a risk factor in asthma with NERD are needed, and it seems important, also among NERD patients, to maintain normal weight in order to decrease the burden of all chronic diseases associated with overweight.

The presence of allergic disease(s) (AR and/or AC and/or AD) was associated with NERD in the unadjusted analysis. In the adjusted analysis, none of the previously listed allergic diseases showed a significant association, but having allergic respiratory symptoms was observed to be a risk factor for NERD. Recent findings support a high prevalence of atopy among NERD patients.^1^ Andersén et al have also listed AR as a risk factor for NERD.^5^ Even though specifically AR was not significant in the adjusted analysis in this study, the results of these articles are in line since they all show an increased risk of atopy and allergic symptoms.

It could have been expected that having nasal polyps and difficult asthma would be risk factors for NERD, since nasal polyps are included in the NERD definition, and it is known that NERD asthmatics tend to have a more severe asthma than non‐NERD asthma patients.^5^, ^6^, ^7^ In our study, self‐reported difficult asthma and nasal polyps were significantly associated with NERD in both unadjusted and adjusted analyses. Our group has also previously shown that NERD is a risk factor for severe asthma,^8^ thus it is not surprising that the correlation works the other way around as well. However, our study did not detect a full association between asthma, CRSwNP and NERD. We have previously demonstrated in a random hospital cohort of rhinitis/CRS patients that there is a strong overlap between these three conditions, and their presence increases the risk of visit frequency,^23^ and the revision sinus surgery of CRS patients.^24^ In the unadjusted analysis, we also found oral corticosteroids regularly or in courses, waking up to symptoms at night several times a month and unable to work for 21 days or in the last year due to respiratory infection(s) to be associated with NERD. This supports the overall idea that NERD is associated with severe or difficult asthma.

The presence of ≥3 older siblings associated with NERD in the adjusted analysis. We have previously demonstrated within this population that the presence of ≥2 siblings was a risk factor for severe adult‐onset asthma.^8^ Although there is growing evidence that early life factors play a role in the development of asthma (i.e. parental smoking, infection, nutrition, rural environment),^25^ whether these early life factors are associated with NERD remains to be addressed. This finding regarding siblings could also be related to infection history. Having many siblings in early childhood could reflect greater infection burden, especially in this population, since in the early and mid‐1900s, children spent a lot of time with their siblings, and children's infections tend to be passed from child to child. Our data allowed us to study larger families than more recent data, as many of the responders were born in a time when families used to have many children.

Interestingly, we found an association between osteoarthritis and NERD. In addition, rheumatoid arthritis and musculoskeletal back disease showed an association in the unadjusted analysis. To our knowledge, this has not been observed previously. Our data include responders from the early 1900s, when Finland was overall more rural, and a greater proportion of people did physically more demanding work nowadays. Physically demanding occupations could be a risk factor for osteoarthritis.^26^ These associations between NERD and comorbidities related to physically demanding work might not be seen with newer data or populations since more and more people are working in less physical occupations. Another hypothesis could be that both NERD and inflammatory joint diseases have shared aetiology related to a history of recurrent bacterial infections and autoinflammation/autoimmunity.^27^ In the early 1900s, fewer people had access to physicians, inflammatory and infectious conditions were not diagnosed and post‐infectious sequelae could develop. Our study found recurrent respiratory infections in early childhood (before school age) to be a risk factor for NERD in the unadjusted analysis, but not in the adjusted analysis. This could have a connection with having many siblings together with putatively an increased genetic risk of recurrent infections.

In this study, we also investigated the effect of adulthood infection history on NERD‐status. NERD was associated with several variables that showed an association in the unadjusted analysis only: the increasing number of feverish flus during the last year, several physician‐diagnosed sinusitis in the last year, several physician‐diagnosed bronchitis in the last year, recurrent respiratory infections in adulthood and having ever had pneumonia. In general, asthma patients have a higher risk for infections.^28^ However, since our study compared non‐NERD‐asthma patients and NERD‐asthma patients, this does not explain the findings. Previous studies suggest that severe asthma increases the risk for certain infections, for example, invasive pneumococcal infection, more than milder asthma.^29^, ^30^ This might explain why NERD‐patients, who tend to have more severe asthma than other asthmatics, seem to be more infection‐prone. NERD‐patients have been identified as prone to middle‐ear infections,^31^ but our study did not find an association between NERD and physician‐diagnosed otitis, which could in part be related to recall bias, as otitis usually occurs in childhood.

Taken together, our results indicate that the association between NERD and inflammatory joint diseases could be related to reactive inflammatory processes in joints and airways, which may have shared immunological aetiology, such as susceptibility to recurrent bacterial airway infections. However, more cohort studies and experimental immunological studies to prove this are still needed.

Eriksson et al. identified current smoking as a risk factor for NERD,^7^ and Andersén et al. identified cumulative exposure to particulate matter (tobacco smoke/work exposure to vapours, gases, dusts and fumes) as a risk factor for NERD.^5^ In this study, ever smoking was not strongly associated with NERD in the adjusted analysis, but exposure to second‐hand smoke at home was associated with NERD in both unadjusted as well as adjusted analyses. NERD patients should avoid exposure to second‐hand smoke. Interestingly, in the adjusted analysis, passive smoking is associated with NERD, but smoking is not, even though smoking has been proposed to be a risk factor for adult‐onset asthma.^32^, ^33^ On the contrary, in the unadjusted analysis, ever smoking seemed to predict a smaller risk for NERD (OR = 0.70, CI = 0.50–0.98, *p* = 0.037). One explanation could be that NERD patients get significant respiratory symptom exacerbations in response to tobacco smoke and thus tend to avoid smoking more often than non‐NERD asthma patients. This would mean that smoking itself would not protect from NERD, but fewer NERD patients are smokers. Indeed, previous studies have shown that smoking asthma patients have poorer disease control compared to non‐smoking asthma patients.^32^, ^33^ However, the prevalences of smoking are relatively close among asthmatics and the general population.^33^ Studies have shown that NERD patients are likely to have more severe asthma than non‐NERD asthma patients.^1^ Considering this, one would imagine it possible that the prevalence of smoking would be smaller among NERD patients than among asthmatics in general since NERD patients would not tolerate tobacco as well. More research is required on the prevalence of smoking among NERD patients and non‐NERD asthma patients to verify this.

Andersen et al. have found that childhood exposure to farming environment affects the probability of childhood‐onset asthma and adult‐onset asthma differently.^34^ Although growing up in a farming environment protects from childhood‐onset asthma, it increases the risk of adult‐onset asthma.^34^ In addition, Andersén et al. have noted that NERD patients were more likely to have been exposed to a farming environment in their childhood than patients with non‐NERD asthma in unadjusted models, but not in multivariable models.^5^ However, in this study, childhood on a farm or in the countryside was not significantly associated with NERD neither in unadjusted nor in adjusted analyses.

This study found recurrent respiratory infections in early childhood (before school age) to be a risk factor for NERD in the unadjusted analysis, but not in the adjusted analysis. This could in theory have a connection with having many siblings, since children easily pass infections to other children, and in the early 1900s young children likely spent a lot of time with their siblings due to less daycare services and long distances, especially in the rural environment, in which the majority of Finnish population were living in the early 1900s. Hence, infections that are typically passed from child to child most probably originated from one's siblings. Prior to this study, it has been observed that respiratory infections in early childhood are associated at least with child‐onset asthma and wheezing,^18^, ^35^, ^36^, ^37^ and this association depends on the number of infections rather than the viral agent. However, adult‐onset asthma is a very different phenotype and conclusions cannot readily be drawn from knowledge on child‐onset asthma. A Finnish study found a strong association between recent respiratory infections and adult‐onset asthma but did not investigate childhood infections.^36^ More research is needed regarding adult‐onset asthma and early childhood infections.

Our study did not detect an association between older age as onset and NERD, whereas Andersén et al. did. Andersén et al. and Eriksson et al. found occupational airborne exposure to be a risk factor for NERD.^5^, ^7^ Our study did not address this, due to limitations of data availability regarding the occupation. Neither did we address visible mould at home, which was observed as a risk factor for NERD in a study by Eriksson et al.^7^ In addition, family history of NERD has been identified as a risk factor,^1^ but this information was not available for us to investigate. Eriksson et al. identified chronic rhinosinusitis as a risk factor for NERD.^7^ We did not have data available regarding chronic rhinosinusitis specifically, but we did address the amount of sinusitis that the patient had in the past year. In our analyses, frequent sinusitis in the past year was associated with NERD in the univariate analysis, but did not show significancy in the multivariable analysis. In a publication by Andersén et al., a family history of asthma and AR was noted to be a risk factor for NERD.^5^ We studied family history of asthma as well, but we did not have information on family history of AR in particular. We investigated if at least one asthmatic parent would be a risk factor for NERD but did not observe an association. One of the differences between our study and Andersén's study is that in our data, the participants were asked only about their parents' asthma status, whereas Andersén's data included siblings' asthma status as well.

When analysing the risk factors of uncontrolled asthma in the NERD and non‐NERD ‐groups, we detected that allergic respiratory symptoms were a common risk factor for uncontrolled asthma in both subgroups. This could reflect Type 2 high diseases as risk factors of severe asthma.^38^ In the non‐NERD group, uncontrolled asthma was also associated with male sex, higher age at asthma onset, musculoskeletal back disease, pneumonia, recurrent bronchitis or other respiratory infections, and oral corticosteroid use, whereas these were not observed in the NERD group. This would imply that NERD patients are a more homogenous group than non‐NERD asthma patients, regardless of their asthma difficulty, and that the risk factors of NERD are unrelated to the difficulty of the disease. However, due to the small number of cases of the NERD subgroup, more studies with an increased number of subjects are still needed.

### 4.1 Strengths and limitations

The strengths of this study include a rather large population‐based data set, which included many NERD patients. In addition, the response rate among asthma patients in the original AAF‐study was high, 84.6%. Since the data dates back to 1997 and includes responders born in the early 1900s, it is possible to study lifestyle factors in the early and mid‐1900s.

The limitations of this study include that we were only able to study some of the factors that could possibly be risk factors of NERD due to limitations of data availability. Furthermore, we acknowledge that the associations with potential risk factors and NERD could be biased since the multivariable analysis does not include all the potential confounders (e.g. occupational exposure) and some of the variables have limited accuracy (i.e. the smoking variable does not take the duration and amount of smoking into account). We acknowledge that we lacked lung function tests or other clinical or biomarker data. We are aware that a small portion of asthmatics might have childhood‐onset asthma that relapsed in adulthood. In addition, a memory bias in the report of risk factors might have occurred. We were not able to study causality between some of the factors, yet as the majority of patients had recently had asthma diagnosis, it can be supposed that the reported factor existed before asthma (and NERD) developed. In this study, NERD status was defined as self‐reported asthma exacerbation due to aspirin, NSAIDs, or general ‘pain reliever’ intake. This is not as exact as physician‐diagnosed NERD. On the other hand, some NERD patients may remain undiagnosed. We acknowledge that many variables used in this study were based on the subjects' opinions and are not fully the same as the clinical definition or diagnostic criteria of a condition, such as NERD. Having a history of respiratory reactions in response to NSAIDs is not a definitive diagnostic method, however, it predicts a positive NERD diagnosis in oral aspirin challenge.^39^ A more accurate diagnosis of NERD would require a challenge test, which may cause risks, require resources and travel as it is available only at the tertiary care. We acknowledge that memory bias may have occurred in our study. In addition, we do not have information about the exact medicines that caused asthma exacerbations in patients who reported exacerbations due to pain relievers. However, NSAIDs, for example, ibuprofen, are widely used as a pain reliever in Finland, thus this kind of presumption is justified. Because of the high response‐rate, asthma diagnosis based on strict reimbursement criteria, and population‐based nature of this study, it could be assumed that the definition of NERD is relatively well covering the asthmatics with NERD. Finally, the interpretation of the results of this study requires some caution, since the questionnaire‐based data date back to 1997. Present and childhood living conditions of current asthma in NERD patients might differ from those of the patients in the AAF‐data, and the prevalence of NERD‐patients may have changed during the last decades. With our age‐stratified analysis, we were able to point out some references on how the risk factors and living conditions might have changed over the decades, but we are aware that absolute conclusions on today's risk factors cannot be drawn based on our study. The youngest patients in our population study were born in the 1960s and Finnish society, living environment and lifestyle have changed since then, which is a limitation in our study. However, studies that were performed with more recent data have obtained fairly similar results to our results regarding risk factors.^5^, ^7^

## 5 CONCLUSION

Our study indicates that female sex, overweight, allergic respiratory symptoms, difficult asthma, nasal polyps, second‐hand smoke exposure at home, having 3 or more older siblings, and osteoarthritis are independently associated with asthma with NERD, and that these factors increase the risk of NERD, except for overweight. Although these results need validation in other populations, in terms of clinical implications, they reinforce the need for smoking cessation in the living environment of asthma with NERD patients, and the importance of early detection and management of risk factors of NERD and other comorbidities to prevent the development of severe adult‐onset asthma with NERD.

## AUTHOR CONTRIBUTIONS

All authors contributed to the planning and conception of the study and the analytical strategy. Alma Helevä, Mikko Nuutinen and Sanna Toppila‐Salmi conducted the data analyses and wrote the manuscript. Alma Helevä, Mikko Nuutinen, Sanna Toppila‐Salmi, and Jussi Karjalainen participated in data curation. Jussi Karjalainen contributed to the collection of the original AAF data. All authors contributed to the analyses, writing and critical review of the manuscript.

## CONFLICT OF INTEREST STATEMENT

ST‐S reports consultancies for ALK‐Abelló, AstraZeneca, ERT, GSK, Novartis, Sanofi, and Roche Products outside the submitted work as well as grant of GSK outside the submitted work. JB reports personal fees from Cipla, Menarini, Mylan, Novartis, Purina, Sanofi‐Aventis, Teva, Uriach, other from KYomed‐Innov, other from Mask‐air‐SAS, outside the submitted work. JK reports personal fees from GlaxoSmithKline, MSD, Novartis, Sanofi, AstraZeneca, Boehringer Ingelheim, Chiesi, and Orion Pharma, outside the submitted work. AL reports personal fees from GlaxoSmithKline and CSL Behring outside the submitted work. All other authors declare no conflicts of interest.

## CONSENT FOR PUBLICATION

Not applicable.

## Supporting information

Tables S1–S2

## Data Availability

Due to Finnish data protection legislation concerning sensitive health information, the datasets generated and/or analysed during this study are not publicly available, and can only be handled by named individuals in the study group for a specific research purpose. The data are available upon reasonable request from the corresponding author. Data use permissions can be applied from the competent authorities.
