# Supplementary material for: Risk factors of NSAID‐exacerbated respiratory disease: A population‐based study
Source: Clin Transl Allergy. 2023 Aug 22;13(8):e12296. doi: 10.1002/clt2.12296 (PMC10442774; doi:10.1002/clt2.12296)
Supplement: Supplementary file 1 — Tables S1–S2 [file CLT2-13-e12296-s001.docx]

**Risk factors of NSAID-exacerbated respiratory disease: A population-based study:**

**Supporting information**

*Table S1: Variables associated with uncontrolled asthma in the group having asthma with NERD (NERD-group).*

|  | Univariate regression model  OR1 (95% CI) | Univariate regression model  p1 | Multivariable regression model  OR2 (95% CI) | Multivariable regression model  p2 |
| --- | --- | --- | --- | --- |
| Sex |  |  |  |  |
| *Male* | 1 |  |  |  |
| *Female* | 0.91 (0.40-2.07) | 0.82 | Not entered |  |
| Underweight (BMI <20) |  |  |  |  |
| *No* | 1 |  |  |  |
| *Yes* | 0.96 (0.23-3.98) | 0.95 | Not entered |  |
| Overweight (BMI >25) |  |  |  |  |
| No | 1 |  |  |  |
| Yes | 1.57 (0.82-2.98) | 0.17 | Not entered |  |
| Age as asthma onset 40 or over |  |  |  |  |
| *No* | 1 |  |  |  |
| *Yes* | 1.17 (0.58-2.37) | 0.67 | Not entered |  |
| Self-reported difficult asthma |  |  |  |  |
| *No* | 1 |  | 1 |  |
| *Yes* | 4.65 (1.77-12.22) | **0.0018** | 4.44 (1.63-12.11) | **0.0036** |
| Oral corticosteroid use regularly or in courses |  |  |  |  |
| *No* | 1 |  |  |  |
| *Yes* | 1.76 (0.91-3.42) | 0.093 | Not entered |  |
| Waking up at night to asthma symptoms several times a month |  |  |  |  |
| *No* | 1 |  |  |  |
| *Yes* | >1000 (0-∞) | 0.99 | Not entered |  |
| ICS and SABA daily |  |  |  |  |
| *No* | 1 |  |  |  |
| *Yes* | >1000 (0-∞) | 0.99 | Not entered |  |
| Nasal polyps |  |  |  |  |
| *No* | 1 |  |  |  |
| *Yes* | 0.59 (0.25-1.36) | 0.22 | Not entered |  |
| Allergic rhinitis |  |  |  |  |
| *No* | 1 |  |  |  |
| *Yes* | 1.77 (0.90-3.47) | 0.099 | Not entered |  |
| Atopic dermatitis |  |  |  |  |
| *No* | 1 |  |  |  |
| *Yes* | 0.61 (0.32-1.15) | 0.13 | Not entered |  |
| Allergic rhinoconjunctivitis |  |  |  |  |
| *No* | 1 |  |  |  |
| *Yes* | 1.55 (0.81-2.95) | 0.19 | Not entered |  |
| Allergic respiratory symptoms |  |  |  |  |
| *No* | 1 |  | 1 |  |
| *Yes* | 2.59 (1.20-5.57) | **0.015** | 2.75 (1.21-6.23) | **0.015** |
| At least one parent with asthma |  |  |  |  |
| *No* | 1 |  |  |  |
| *Yes* | 0.76 (0.40-1.46) | 0.41 | Not entered |  |
| Osteoarthritis |  |  |  |  |
| *No* | 1 |  |  |  |
| *Yes* | 1.24 (0.62-2.47) | 0.54 | Not entered |  |
| Back disease |  |  |  |  |
| *No* | 1 |  |  |  |
| *Yes* | 1.81 (0.92-3.56) | 0.085 | Not entered |  |
| Rheumatoid arthritis |  |  |  |  |
| *No* | 1 |  |  |  |
| *Yes* | 1.97 (0.57-6.85) | 0.29 | Not entered |  |
| Hypertension medication |  |  |  |  |
| *No* | 1 |  |  |  |
| *Yes* | 1.76 (0.83-3.75) | 0.14 | Not entered |  |
| Recurrent respiratory infections before school age |  |  |  |  |
| *No* | 1 |  |  |  |
| *Yes* | 1.48 (0.57-3.85) | 0.42 | Not entered |  |
| Recurrent respiratory infections in school age |  |  |  |  |
| *No* | 1 |  |  |  |
| *Yes* | 1.42 (0.67-3.03) | 0.36 | Not entered |  |
| Recurrent respiratory infections in adulthood |  |  |  |  |
| *No* | 1 |  |  |  |
| *Yes* | 1.41 (0.70-2.82) | 0.33 | Not entered |  |
| Severe infections in childhood |  |  |  |  |
| *No* | 1 |  |  |  |
| *Yes* | 2.17 (0.94-5.01) | 0.070 | Not entered |  |
| Pneumonia ever |  |  |  |  |
| *No* | 1 |  | 1 |  |
| *Yes* | 2.34 (1.20-4.58) | **0.013** | 2.03 (1.00-4.12) | 0.051 |
| Physician-diagnosed pneumonia in the last year |  |  |  |  |
| *No* | 1 |  |  |  |
| *Yes* | >1000 (0-∞) | 0.99 | Not entered |  |
| Physician-diagnosed otitis in the last year |  |  |  |  |
| *No* | 1 |  |  |  |
| *Yes* | 0.74 (0.19-2.85) | 0.66 | Not entered |  |
| Physician-diagnosed angina in the last year |  |  |  |  |
| *No* | 1 |  |  |  |
| *Yes* | 0 (0-∞) | 0.99 | Not entered |  |
| Physician-diagnosed sinuitis in the last year |  |  |  |  |
| *0/unknown* | 1 |  |  |  |
| *1-2* | 1.92 (0.79-4.66) | 0.15 |  |  |
| *3+* | 0.68 (0.18-2.53) | 0.56 | Not entered |  |
| Physician-diagnosed bronchitis in the last year |  |  |  |  |
| *0/unknown* | 1 |  |  |  |
| *1-2* | 1.00 (0.49-2.02) | 1.00 |  |  |
| *3+* | >1000 (0-∞) | 0.99 | Not entered |  |
| Feverish flu in the last year |  |  |  |  |
| *0/unknown* | 1 |  |  |  |
| *1-3* | 1.04 (0.53-2.01) | 0.92 |  |  |
| *4+* | 5.00 (0.56-44.52) | 0.15 | Not entered |  |
| Unable to work for 21d or over in the past year due to respiratory infection(s) |  |  |  |  |
| *No* | 1 |  |  |  |
| *Yes* | 1.93 (0.77-4.87) | 0.16 | Not entered |  |
| Smoking ever |  |  |  |  |
| *No* | 1 |  |  |  |
| *Yes* | 0.71 (0.38-1.34) | 0.29 | Not entered |  |
| Smoker parent(s) |  |  |  |  |
| *No* | 1 |  |  |  |
| *Yes* | 1.48 (0.78-2.80) | 0.23 | Not entered |  |
| Second-hand tobacco smoke exposure at home |  |  |  |  |
| *No* | 1 |  |  |  |
| *Yes* | 0.79 (0.25-2.46) | 0.68 | Not entered |  |
| 4. or later child |  |  |  |  |
| *No* | 1 |  |  |  |
| *Yes* | 1.25 (0.65-2.39) | 0.50 | Not entered |  |
| Childhood spent on a farm |  |  |  |  |
| *No* | 1 |  |  |  |
| *Yes* | 1.43 (0.75-2.71) | 0.27 | Not entered |  |
| Childhood spent on countryside |  |  |  |  |
| *No* | 1 |  |  |  |
| *Yes* | 0.83 (0.40-1.72) | 0.62 | Not entered |  |

ICS = inhaled corticosteroids; SABA = short-acting beta agonists. OR = odds ratio. CI= confidence interval. p = p-value. p-value <0.05 was considered statistically significant. The variables with p<0.05 in the univariate analysis were entered in the multivariable analysis. The total number of subjects having asthma with NERD was 153. The number of subjects having uncontrolled asthma with NERD was 79 (51.6%) and those with controlled asthma with NERD was 74 (48.4%). Uncontrolled asthma was defined as having wake-ups at night due to asthma symptoms several times a month (N=44), or daily ICS and SABA use (N=11), or both (N=24).

*Table S2: Variables associated with uncontrolled asthma in the group having asthma without NERD (non-NERD -group)*

|  | Univariate regression model  OR1 (95% CI) | Univariate regression model  p1 | Multivariable regression model  OR2 (95% CI) | Multivariable regression model  p2 |
| --- | --- | --- | --- | --- |
| Sex |  |  |  |  |
| *Male* | 1 |  | 1 |  |
| *Female* | 0.52 (0.41-0.66) | **<0.001** | 0.55 (0.42-0.72) | **<0.001** |
| Underweight (BMI <20) |  |  |  |  |
| *No* | 1 |  |  |  |
| *Yes* | 0.73 (0.42-1.29) | 0.28 | Not entered |  |
| Overweight (BMI >25) |  |  |  |  |
| No | 1 |  | 1 |  |
| Yes | 1.33 (1.04-1.70) | **0.021** | 1.21 (0.92-1.59) | 0.17 |
| Age as asthma onset 40 or over |  |  |  |  |
| *No* | 1 |  | 1 |  |
| *Yes* | 1.95 (1.48-2.58) | **<0.001** | 1.92 (1.40-2.63) | **<0.001** |
| Self-reported difficult asthma |  |  |  |  |
| *No* | 1 |  | 1 |  |
| *Yes* | 7.30 (4.49-11.86) | **<0.001** | 5.08 (3.01-8.59) | **<0.001** |
| Oral corticosteroid use regularly or in courses |  |  |  |  |
| *No* | 1 |  | 1 |  |
| *Yes* | 2.64 (2.04-3.42) | **<0.001** | 1.93 (1.44-2.59) | **<0.001** |
| Waking up at night to asthma symptoms several times a month |  |  |  |  |
| *No* | 1 |  |  |  |
| *Yes* | >1000 (0-∞) | 0.97 | Not entered |  |
| ICS and SABA daily |  |  |  |  |
| *No* | 1 |  |  |  |
| *Yes* | >1000 (0-∞) | 0.96 | Not entered |  |
| Nasal polyps |  |  |  |  |
| *No* | 1 |  |  |  |
| *Yes* | 1.03 (0.71-1.50) | 0.87 | Not entered |  |
| Allergic rhinitis |  |  |  |  |
| *No* | 1 |  |  |  |
| *Yes* | 0.84 (0.67-1.06) | 0.14 | Not entered |  |
| Atopic dermatitis |  |  |  |  |
| *No* | 1 |  |  |  |
| *Yes* | 0.81 (0.63-1.04) | 0.092 | Not entered |  |
| Allergic rhinoconjunctivitis |  |  |  |  |
| *No* | 1 |  |  |  |
| *Yes* | 1.03 (0.82-1.30) | 0.80 | Not entered |  |
| Allergic respiratory symptoms |  |  |  |  |
| *No* | 1 |  | 1 |  |
| *Yes* | 1.34 (1.07-1.68) | **0.012** | 1.30 (1.00-1.69) | **0.0496** |
| At least one parent with asthma |  |  |  |  |
| *No* | 1 |  |  |  |
| *Yes* | 0.79 (0.62-1.01) | 0.057 | Not entered |  |
| Osteoarthritis |  |  |  |  |
| *No* | 1 |  |  |  |
| *Yes* | 1.34 (0.99-1.83) | 0.058 | Not entered |  |
| Back disease |  |  |  |  |
| *No* | 1 |  | 1 |  |
| *Yes* | 1.57 (1.21-2.04) | **<0.001** | 1.38 (1.03-1.84) | **0.029** |
| Rheumatoid arthritis |  |  |  |  |
| *No* | 1 |  |  |  |
| *Yes* | 0.82 (0.46-1.49) | 0.52 | Not entered |  |
| Hypertension medication |  |  |  |  |
| *No* | 1 |  |  |  |
| *Yes* | 1.24 (0.94-1.63) | 0.13 | Not entered |  |
| Recurrent respiratory infections before school age |  |  |  |  |
| *No* | 1 |  |  |  |
| *Yes* | 0.89 (0.55-1.45) | 0.64 | Not entered |  |
| Recurrent respiratory infections in school age |  |  |  |  |
| *No* | 1 |  |  |  |
| *Yes* | 0.98 (0.74-1.32) | 0.92 | Not entered |  |
| Recurrent respiratory infections in adulthood |  |  |  |  |
| *No* | 1 |  | 1 |  |
| *Yes* | 1.60 (1.26-2.02) | **<0.001** | 1.49 (1.14-1.96) | **0.0040** |
| Severe infections in childhood |  |  |  |  |
| *No* | 1 |  | 1 |  |
| *Yes* | 1.36 (1.00-1.85) | **0.048** | 1.36 (0.97-1.90) | 0.076 |
| Pneumonia ever |  |  |  |  |
| *No* | 1 |  |  |  |
| *Yes* | 1.25 (0.97-1.60) | 0.086 | Not entered |  |
| Physician-diagnosed pneumonia in the last year |  |  |  |  |
| *No* | 1 |  | 1 |  |
| *Yes* | 3.72 (1.56-8.87) | **0.0030** | 2.62 (1.04-6.58) | **0.040** |
| Physician-diagnosed otitis in the last year |  |  |  |  |
| *No* | 1 |  |  |  |
| *Yes* | 1.23 (0.71-2.14) | 0.46 | Not entered |  |
| Physician-diagnosed angina in the last year |  |  |  |  |
| *No* | 1 |  |  |  |
| *Yes* | 1.56 (0.76-3.19) | 0.23 | Not entered |  |
| Physician-diagnosed sinuitis in the last year |  |  |  |  |
| *0/unknown* | 1 |  |  |  |
| *1-2* | 1.03 (0.72-1.47) | 0.88 |  |  |
| *3+* | 1.39 (0.63-3.07) | 0.42 | Not entered |  |
| Physician-diagnosed bronchitis in the last year |  |  |  |  |
| *0/unknown* | 1 |  | 1 |  |
| *1-2* | 1.92 (1.42-2.61) | **<0.001** | 1.56 (1.11-2.20) | **0.011** |
| *3+* | 3.06 (1.56-6.00) | **0.0012** | 2.07 (0.97-4.42) | 0.059 |
| Feverish flu in the last year |  |  |  |  |
| *0/unknown* | 1 |  |  |  |
| *1-3* | 0.99 (0.77-1.28) | 0.96 |  |  |
| *4+* | 1.63 (0.73-3.62) | 0.23 | Not entered |  |
| Unable to work for 21d or over in the past year due to respiratory infection(s) |  |  |  |  |
| *No* | 1 |  | 1 |  |
| *Yes* | 1.78 (1.19-2.66) | **0.0053** | 1.02 (0.65-1.61) | 0.93 |
| Smoking ever |  |  |  |  |
| *No* | 1 |  | 1 |  |
| *Yes* | 1.56 (1.23-1.97) | **<0.001** | 1.17 (0.89-1.54) | 0.26 |
| Smoker parent(s) |  |  |  |  |
| *No* | 1 |  |  |  |
| *Yes* | 1.10 (0.88-1.39) | 0.40 | Not entered |  |
| Second-hand tobacco smoke exposure at home |  |  |  |  |
| *No* | 1 |  |  |  |
| *Yes* | 1.39 (0.80-2.42) | 0.25 | Not entered |  |
| 4. or later child |  |  |  |  |
| *No* | 1 |  |  |  |
| *Yes* | 1.02 (0.79-1.32) | 0.87 | Not entered |  |
| Childhood spent on a farm |  |  |  |  |
| *No* | 1 |  |  |  |
| *Yes* | 1.10 (0.87-1.39) | 0.42 | Not entered |  |
| Childhood spent on countryside |  |  |  |  |
| *No* | 1 |  |  |  |
| *Yes* | 1.23 (0.95-1.61) | 0.12 | Not entered |  |

ICS = inhaled corticosteroids; SABA = short-acting beta agonists. OR = odds ratio. CI= confidence interval. p = p-value. p-value <0.05 was considered statistically significant. The variables with p<0.05 in the univariate analysis were entered in the multivariable analysis. The total number of subjects having non-NERD asthma was 1197. The number of subjects having uncontrolled non-NERD asthma was 528 (44.1%) and those with controlled non-NERD asthma was 669 (55.9%). Uncontrolled asthma was defined as having wake-ups at night due to asthma symptoms several times a month (N=287), or daily ICS and SABA use (N=118), or both (N=123).
